# Tracing neurovascular pathways: a conversation with Martin Lauritzen

**DOI:** 10.1117/1.NPh.13.3.030401

**Published:** 2026-07-09

**Authors:** Changsi Cai

**Affiliations:** University of Copenhagen, Department of Neuroscience, Copenhagen, Denmark

## Abstract

Martin Lauritzen, Professor of Neuroscience at University of Copenhagen, discusses his career in a conversation with his colleague and former mentee, Changsi Cai, Associate Editor for Neurophotonics.

**Figure:**
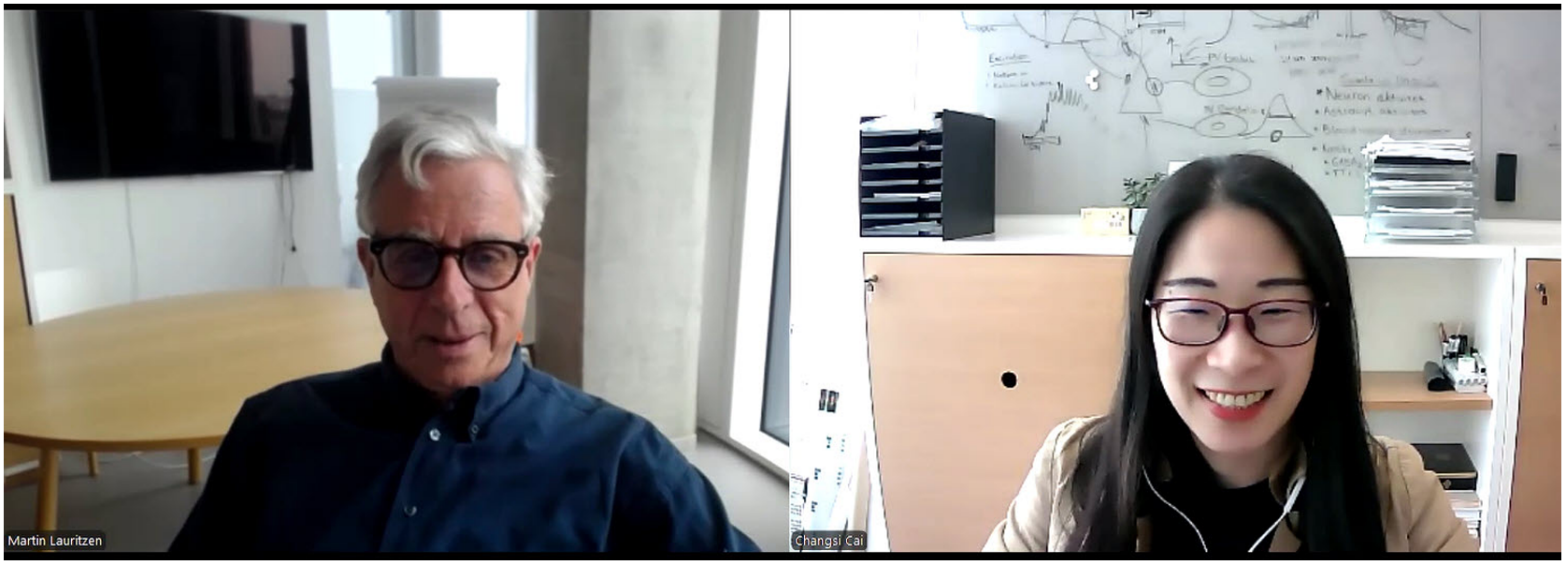
*Neurophotonics* Associate Editor Changsi Cai (right) interviewed Martin Lauritzen (left), Professor of Neuroscience at University of Copenhagen. Lauritzen has played a central role in defining how neural activity, blood flow, and metabolism interact in health and disease. In this interview, he reflects on how his research trajectory unfolded, the observations that changed his thinking, and why aging and drug delivery now reside at the center of his work. View the interview video here: https://doi.org/10.1117/1.NPh.13.3.030401.

Martin Lauritzen’s career has followed one central question: how brain activity, blood flow, and metabolism work together in health and disease. Trained as a physician and shaped by both clinical practice and experimental research, he has helped clarify how vascular signals reflect—and sometimes drive—brain function. In this conversation, Lauritzen reflects on the discoveries that shaped his work and on why aging and drug delivery are now key priorities.

Lauritzen did not start out in neuroscience. As a medical student, he worked on protein chemistry in the kidney, gaining a strong biochemical grounding. His path shifted during a neurology internship, where he encountered early human brain imaging based on cerebral blood flow. Seeing language and sensory functions mapped through physiology convinced him that blood flow measurements could reveal brain activity directly.

That insight led him to migraine research. While studying cerebral blood flow during migraine attacks, Lauritzen observed a slow, spreading reduction in blood flow that did not follow the pattern of major arteries. The finding pointed to processes in the brain’s microvasculature. At the same time, similarities between these vascular changes and cortical spreading depression—a wave of neuronal depolarization seen in experimental models—became apparent. Moving into experimental physiology, Lauritzen helped show that spreading depression causes marked blood flow reductions, linking migraine aura to this mechanism. Once controversial, this idea is now widely accepted.

Alongside this work, Lauritzen became deeply involved in functional brain imaging. When fMRI was introduced, its signals were widely used but poorly understood. By combining electrophysiology with vascular measurements, his group showed that increases in blood flow track synaptic activity rather than neuronal firing. These responses depended on glutamatergic signaling and nitric oxide, challenging simple metabolic interpretations of imaging signals and helping establish the physiological basis of neurovascular coupling.

A major turning point came with *in vivo* two-photon microscopy. For the first time, Lauritzen’s lab could directly observe cellular and vascular processes in the living brain. This work clarified that calcium signaling is critical for driving blood flow, while oxygen consumption more closely reflects synaptic currents. The approach also allowed his team to revisit earlier questions about regional differences and disease-related changes in neurovascular signaling.

Lauritzen later brought his long-standing interest in spreading depolarization back to the clinic. Working with neurosurgeons, he recorded these waves directly from the brains of patients with traumatic injury or ischemia. The results showed that spreading depolarizations are common in injured brains and are linked to worse outcomes, reframing them as drivers of secondary damage rather than passive byproducts.

More recently, Lauritzen’s focus has shifted to aging and vulnerability. His work suggests that as the brain ages, its physiological “reserve” declines: blood vessels stiffen, responses weaken, and the margin for handling stress narrows—often beginning earlier in adulthood than commonly assumed. This line of thinking naturally extends to his current research on the blood–brain barrier and drug delivery, an urgent challenge as therapies increasingly rely on large biological molecules. Using high-resolution imaging, his group aims to map how transport across the barrier changes in health and disease.

Looking back, Lauritzen highlights moments where new tools or perspectives changed his thinking, but he also emphasizes the value of sustained, collaborative work. For early-career scientists, he points to drug delivery and small-vessel disease as areas likely to shape the future of neuroscience. His career underscores a simple lesson: following physiology across boundaries often leads to the most enduring insights.

